# Frail by different measures: a comparison of 8-year mortality in The Irish Longitudinal Study on Ageing (TILDA)

**DOI:** 10.1007/s41999-021-00570-9

**Published:** 2021-11-01

**Authors:** Roman Romero-Ortuno, Peter Hartley, Rose Anne Kenny, Aisling M. O’Halloran

**Affiliations:** 1 The Irish Longitudinal Study on Ageing (TILDA), Trinity College, Dublin, Ireland; 2 Discipline of Medical Gerontology, School of Medicine, Trinity College, Dublin, Ireland; 3 Mercer’s Institute for Successful Ageing, St James’s Hospital, Dublin, Ireland; 4 Department of Public Health and Primary Care, University of Cambridge, Cambridge, UK

**Keywords:** Frailty, Mortality, Comparative study, Longitudinal study, Older people

## Abstract

**Purpose:**

We compared the ability of four frailty identification tools (frailty phenotype: FP; FRAIL scale; 32-item Frailty Index: FI; and Clinical Frailty Scale: CFS) to predict 8-year mortality in TILDA.

**Methods:**

We included wave 1 (2010) participants with data for all four tools. Mortality was ascertained at wave 5 (2018). Age, sex and education-adjusted binary logistic regression models were computed.

**Results:**

At baseline, there were 5700 participants (mean age 63, range 50–98, 54% women). Frailty prevalences were 2.3% by FRAIL, 3.8% by FP, 10.9% by CFS, and 12.8% by FI. Mortality was 41.2%, 44.9%, 25.3% and 27.0%, respectively. The highest adjusted OR for mortality was for FRAIL (OR 4.48, 95% CI 2.93–6.85, *P* < 0.001), followed by FP (OR 3.55, 95% CI 2.52–5.00, *P* < 0.001), FI (OR 2.10, 95% CI 1.68–2.62, *P* < 0.001), and CFS (OR 1.88, 95% CI 1.48–2.38, *P* < 0.001).

**Conclusions:**

All tools significantly predicted mortality, but FRAIL and FP seemed more specific.

## Key summary points

**Aim:**

To compare how four different frailty classifications predicted 8-year mortality in TILDA.

**Findings:**

In those aged 65 or more years, frailty prevalences were 3.7% by FRAIL, 6.7% by FP, 16.6% by CFS, and 22.0% by FI. Mortality proportions were 57.1%, 57.8%, 36.8% and 35.6%, respectively.

**Message:**

All tools significantly predicted mortality, but FRAIL and FP seemed more specific.

## Introduction

Frailty is a state of dysregulation in multiple physiological systems and vulnerability to stressors [1]. Many frailty identification tools exist that differ in their conceptualisation vis-à-vis morbidity, disability, cognition, and other geriatric assessment dimensions [2].

Several population-based studies have compared frailty identification tools for the prediction of mortality, including the Health and Retirement Study (HRS) [3] and the National Health and Nutrition Examination Survey (NHANES) [4] in the USA; and, in Europe, the Survey of Health, Ageing and Retirement in Europe (SHARE) [5], the Tree-City study [6], and the English Longitudinal Study of Ageing (ELSA) [7]. These are among the many studies that have compared the performance of different frailty tools in their prediction of adverse health-related outcomes in different populations.

The present study added value by comparing the ability of four different frailty identification tools to predict 8-year mortality in The Irish Longitudinal Study on Ageing (TILDA).

## Methods

### Design and setting

We analysed data from TILDA, a population-based longitudinal study that collects information on the health, economic and social circumstances from people aged 50 and over in Ireland. Wave 1 of the study (baseline) took place between October 2009 and February 2011, and subsequent data were collected approximately 2-yearly over four longitudinal waves (wave 2: February 2012 to March 2013; wave 3: March 2014 to October 2015; wave 4: January to December 2016; wave 5: January to December 2018). The full cohort profile has been described elsewhere [8]. All performance-based health measures in TILDA were collected by trained research nurses following standard operating procedures.

### Participants

We included TILDA wave 1 participants aged 50 years or over who had complete data for frail state classification according to four frailty identification tools.

### Measures

Frail state was defined as per the following four tools:

- Fried’s physical frailty phenotype (FP): frail if ≥ 3 features present. The operationalisation of the frailty phenotype in TILDA was the same as in the Cardiovascular Health Study [9], except for the physical activity criterion, for which we used the short form of the International Physical Activity Questionnaire (IPAQ) [10].
- Morley’s FRAIL scale (frail if ≥ 3 among fatigue, resistance, ambulation, illnesses, and loss of weight). This was operationalised in TILDA as previously described [11].
- A 32-item Frailty Index (FI ≥ 0.25), the description of which has been detailed elsewhere [12],
- and the Clinical Frailty Scale classification tree (CFS ≥ 5), as detailed elsewhere [13].

Age, sex and education were collected at baseline. The latter was defined as a three-level ordinal variable: up to primary (reference category), secondary and higher.

Mortality was ascertained for all study participants at each follow-up wave, following procedures described elsewhere [14].

### Statistical analyses

All statistical analyses were carried out using IBM SPSS Statistics for Windows (Version 26.0. Armonk, NY: IBM Corp). Descriptives were given as mean with standard deviation (SD) and range, or count with percentage (%). Overlap between the four frailty classifications was visualised by means of a Venn diagram created with the ggvenn package in R (version 0.1.9; https://CRAN.R-project.org/package=ggvenn). Sensitivity and specificity for 8-year mortality were calculated for each frailty tool using MedCalc Software Ltd. Diagnostic test evaluation calculator, https://www.medcalc.org/calc/diagnostic_test.php (Version 20.009; accessed August 3, 2021). In addition, binary logistic regression models controlling for age, sex and education were computed for the extraction of odds ratios (OR) and 95% confidence intervals (CI). We did not control those models for multimorbidity or baseline physical activity because these were already included in some frailty definitions. The level of statistical significance was set at *p* < 0.05. A sensitivity analysis was performed on the baseline sample aged 65 and over.

### Ethics

Ethical approval for each wave was obtained from the Faculty of Health Sciences Research Ethics Committee at Trinity College Dublin, Ireland. All participants provided written informed consent.

## Results

**Fig. 1:**
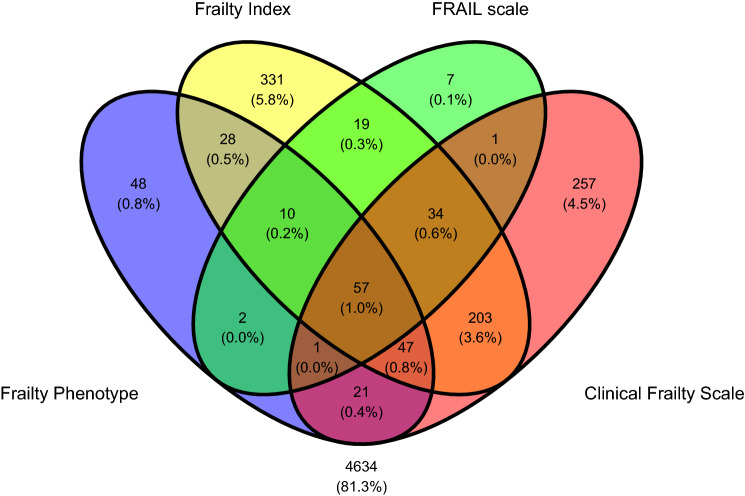
Venn diagram representing the overlap between the four frailty classifications in the first wave of TILDA (population aged 50 and over, *N* = 5700). Number and percentages of TILDA wave 1 participants are shown. 4634 participants were not classified as frail by any scheme

**Fig. 2:**
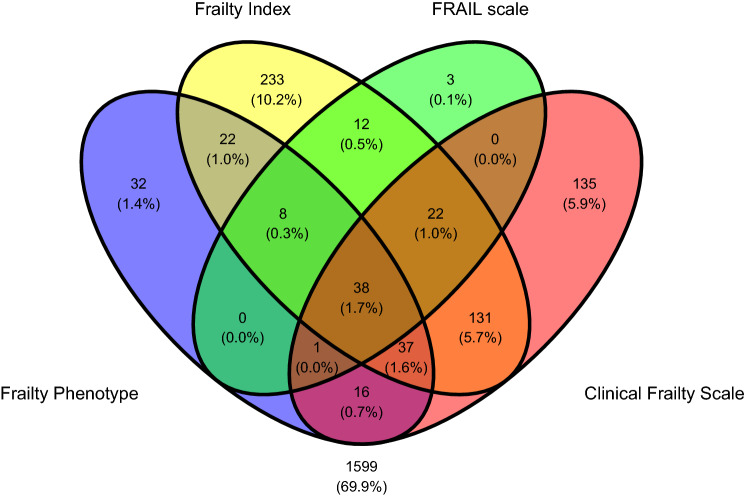
Venn diagram representing the overlap between the four frailty classifications in the first wave of TILDA (population aged 65 and over, *N* = 2289). Number and percentages of TILDA wave 1 participants are shown. 1599 participants were not classified as frail by any scheme

At wave 1, there were 5700 participants (mean age 63, SD 9.3, range 50–98 years, 54% women, 26% with primary education or less, and 33% with higher education) with data for frailty classification according to all four tools. The prevalences of frailty were 2.3% (*N* = 131) by FRAIL, 3.8% (*N* = 214) by FP, 10.9% (*N* = 621) by CFS, and 12.8% (*N* = 729) by FI. Only 57 participants (1%) were classified as frail by all tools. The vast majority of wave 1 participants (4634 or 81.3%) were not classified as frail by any of the tools. Figure 1 shows the overlap between the four frailty classifications. Figure 2 shows the overlap in those aged 65 and over.

The 8-year mortality proportions (in those aged ≥ 50) were 44.9% by FP, 41.2% by FRAIL, 27.0% by FI, and 25.3% by CFS. The 1% classified as frail by all tools had a mortality proportion of 43.9%. The mortality for the 81.3% not classified as frail by any tool was 7.0% (Table 1). Table 2 shows the proportions for those aged ≥ 65.

**Table 1:** Comparison of 8-year mortality proportions and binary logistic regression results (population aged 50 and over, *N* = 5700)

	% 8-year mortality	OR (age, sex, and education-adjusted)	Lower 95% CI for OR	Upper 95% for OR	*P*
Frail by FRAIL (*N* = 131)	41.2	4.48	2.93	6.85	< 0.001
Frail by FP (*N* = 214)	44.9	3.55	2.52	5.00	< 0.001
Frail by FI (*N* = 729)	27.0	2.10	1.68	2.62	< 0.001
Frail by CFS (*N* = 621)	25.3	1.88	1.48	2.38	< 0.001
Frail by all (*N* = 57)	43.9	3.92	2.05	7.50	< 0.001
Frail by none (*N* = 4634)	7.0	0.46	0.38	0.57	< 0.001

*OR* odds ratio, *CI* confidence interval, *FP* frailty phenotype, *FI* Frailty Index, *CFS* Clinical Frailty Scale

**Table 2:** Comparison of 8-year mortality proportions and binary logistic regression results (population aged 65 and over, *N* = 2289)

	% 8-year mortality	OR (age, sex, and education-adjusted)	Lower 95% CI for OR	Upper 95% for OR	*P*
Frail by FRAIL (*N* = 84)	57.1	5.07	3.08	8.34	< 0.001
Frail by FP (*N* = 154)	57.8	3.47	2.38	5.06	< 0.001
Frail by FI (*N* = 503)	35.6	2.12	1.66	2.72	< 0.001
Frail by CFS (*N* = 380)	36.8	1.83	1.40	2.39	< 0.001
Frail by all (*N* = 38)	63.2	4.79	2.28	10.09	< 0.001
Frail by none (*N* = 1599)	14.9	0.47	0.37	0.60	< 0.001

The calculated sensitivities, specificities and associated diagnostic statistics for 8-year mortality according to each frailty tool are shown in Table 3 (aged ≥ 50). In general, frailty classifications had high specificity, that is, the probabilities of the tests predicting survival when baseline frailty was not identified were high (from highest to lowest: FRAIL 98.5%, FP 97.7%, CFS 90.9% and FI 89.6%). However, sensitivities (probability of the test predicting death when frailty was identified) were generally low (from highest to lowest: FI, 33.3%, CFS 26.6%, FP 16.2% and FRAIL 9.1%). Adjusted by the corresponding frailty prevalences, negative predictive values were high (and ordered the same as specificities from highest to lowest: FRAIL 97.9%, FP 96.7%, CFS 91.0% and FI 90.2%), and positive predictive values were low (and ordered the same as sensitivities from highest to lowest: FI, 32.0%, CFS 26.4%, FP 21.7% and FRAIL 12.5%). In terms of accuracy (overall probability that a participant was correctly classified), values were, from highest to lowest: FRAIL 96.4%, FP 94.6%, CFS 83.9%, and FI 82.4%. Table 4 shows the results for those aged ≥ 65.

**Table 3:** Diagnostic evaluation of the four frail classifications for the prediction of 8-year mortality in TILDA (population aged 50 and over, *N* = 5700)

	Value	95% CI	Value	95% CI
	FRAIL		Frailty phenotype	
Sensitivity	9.14%	6.94–11.75%	16.24%	13.36–19.47%
Specificity	98.49%	98.12–98.81%	97.69%	97.24–98.08%
Positive likelihood ratio	6.06	4.33–8.49	7.03	5.45–9.08
Negative likelihood ratio	0.92	0.90–0.95	0.86	0.83–0.89
Frailty prevalence	2.30%		3.80%	
Positive predictive value	12.49%	9.24–16.66%	21.74%	17.71–26.40%
Negative predictive value	97.87%	97.82–97.93%	96.72%	96.61–96.84%
Accuracy	96.44%	95.92–96.90%	94.60%	93.98–95.17%

	Clinical frailty scale		Frailty index	
Sensitivity	26.57%	23.04–30.32%	33.33%	29.54–37.29%
Specificity	90.92%	90.10–91.69%	89.59%	88.72–90.41%
Positive likelihood ratio	2.93	2.49–3.43	3.2	2.78–3.68
Negative likelihood ratio	0.81	0.77–0.85	0.74	0.70–0.79
Frailty prevalence	10.90%		12.80%	
Positive predictive value	26.35%	23.37–29.57%	31.97%	29.01–35.08%
Negative predictive value	91.01%	90.60–91.40%	90.15%	89.63–90.65%
Accuracy	83.90%	82.92–84.85%	82.39%	81.37–83.37%

**Table 4:** Diagnostic evaluation of the four frail classifications for the prediction of 8-year mortality in TILDA (population aged 65 and over, *N* = 2289)

	Value	95% CI	Value	95% CI
	FRAIL		Frailty phenotype	
Sensitivity	10.08%	7.53–13.15%	18.70%	15.29–22.50%
Specificity	98.01%	97.26–98.61%	96.41%	95.45–97.22%
Positive likelihood ratio	5.08	3.34–7.73	5.22	3.85–7.06
Negative likelihood ratio	0.92	0.89–0.95	0.84	0.81–0.88
Frailty prevalence	3.70%		6.70%	
Positive predictive value	16.33%	11.36–22.90%	27.25%	21.66–33.66%
Negative predictive value	96.60%	96.49–96.70%	94.29%	94.05–94.52%
Accuracy	94.76%	93.77–95.64%	91.21%	89.97–92.34%

	Clinical frailty scale		Frailty index	
Sensitivity	29.41%	25.35–33.73%	37.61%	33.24–42.13%
Specificity	86.76%	85.12–88.29%	82.13%	80.29– 83.87%
Positive likelihood ratio	2.22	1.85–2.67	2.1	1.81–2.45
Negative likelihood ratio	0.81	0.77–0.86	0.76	0.71–0.82
Frailty prevalence	16.60%		22.00%	
Positive predictive value	30.66%	26.93–34.67%	37.25%	33.76–40.86%
Negative predictive value	86.06%	85.32–86.78%	82.35%	81.27–83.39%
Accuracy	77.24%	75.47–78.95%	72.33%	70.45–74.16%

Calculations performed in Software Ltd. Diagnostic test evaluation calculator. https://www.medcalc.org/calc/diagnostic_test.php (Version 20.009; accessed September 11, 2021)

Table 1 shows the result of the binary logistic regression models in those aged ≥ 50. The highest adjusted OR for mortality was for FRAIL (OR 4.48, 95% CI 2.93–6.85, *P* < 0.001), followed by FP (OR 3.55, 95% CI 2.52–5.00, *P* < 0.001), FI (OR 2.10, 95% CI 1.68–2.62, *P* < 0.001), and CFS (OR 1.88, 95% CI 1.48–2.38, *P* < 0.001). Table 2 shows the results for those aged ≥ 65.

## Discussion

In the present study, we compared the ability of four different frailty identification tools to predict 8-year mortality in TILDA. All four frailty tools significantly predicted 8-year mortality, but FRAIL and FP seemed more specific and FI and CFS a little more sensitive, although sensitivities were generally very low. Consequently, in the context of a population-based study where the general mortality proportion and prevalences of frailty are low, the use of a frailty identification tool for the specific purpose of the prediction of 8-year mortality could be useful *to rule out the outcome* when frailty is not identified at baseline, rather than *to predict the outcome* when frailty is identified. In this regard, the most accurate tool to rule out 8-year mortality was the negative classification by FRAIL, followed by the negative classification by FP (Tables 3 and 4).

Our results are in keeping with those of other population-based studies. In HRS, researchers cross-sectionally compared three models of frailty (functional domains, burden model and biologic syndrome), and also noted that a very small proportion of participants (3.1%) were frail according to all three models, with a significant overlap in the proportions of participants classified as frail [3]. In NHANES, researchers also found that the prevalence of frailty was lower using the FP approach (3.6%) compared to the FI (34%) [4]. In SHARE, researchers found areas under the curve (AUC) for the prediction of 5-year mortality of 0.67 (95% CI 0.65–0.68) for the FRAIL scale, 0.70 (0.68–0.71) for the FP, 0.70 (0.68–0.71) for the CFS, and 0.75 (0.74–0.77) for the FI, which is consistent with our result that FRAIL had the lowest and FI the highest PPV, although in their study AUCs for FP and CFS were indistinguishable [5]. In the Tree-City study, the 10-year mortality AUC was also lower for FP (0.56 [0.52–0.60]) than for FI (0.63 [0.60–0.67]) [6]. In a large comparative study, ELSA researchers noted that fully adjusted 3.5-year mortality hazard ratios (HRs) for frailty states varied depending on the classification used: 1.5 (95% CI 0.8–3.0) for FRAIL, 1.8 (0.7–4.4) for FP, and 2.3 (1.5–3.5) for a CGA-based FI [7], also in keeping with our PPV results.

Given that the gold standard for the assessment and management of frailty in an individual is the provision of comprehensive geriatric assessment (CGA), which is time and resource intensive, it would appear reasonable (from the simplistic perspective of aiming to lower the 8-year mortality risk in the population) that a negative result in either FRAIL or FP would be used *to rule out CGA referral*, and a positive result in any tool used *to rule in CGA referral*, with a prioritisation as follows according to positive predictive values (PPV), from most to least urgent: FI, CFS, FP and FRAIL (this order of decreasing PPV values applies to both ≥ 50 and ≥ 65 populations as per Tables 3 and 4). Naturally, this recommendation is more theoretical than practical, because CGA indication is not only the reduction of mortality risk, but also the preservation of functional decline and promotion of independent living [15]. In addition, this theoretical scheme could be difficult to implement in real practice since we found overlaps in the proportions of participants classified as frail by the four tools (Figs. 1 and 2). However, the results of our study help appreciate that different frailty tools have different diagnostic properties and could be used differently in population screening programmes and clinical pathways.
